# Relationship between Sleep and Psychosis in the Pediatric Population: A Brief Review

**DOI:** 10.3390/medsci6030076

**Published:** 2018-09-14

**Authors:** Meelie Bordoloi, Ujjwal Ramtekkar

**Affiliations:** 1 Department of Psychiatry, University of Missouri, Columbia, MO 65212, USA; bordoloim@health.missouri.edu; 2 Department of Psychiatry, Nationwide Children’s Hospital, Columbus, OH 43025, USA

**Keywords:** sleep disturbance, psychosis, schizophrenia, pediatric sleep

## Abstract

Sleep disorders are common in several psychiatric disorders, including schizophrenia. In the pediatric population, the relationship between sleep and psychosis is not completely understood due to limited research studies investigating the link. Insomnia is noted to be a predictor of psychosis, especially in ultrahigh risk adolescents. Sleep difficulties are also associated with a two to three-fold increase in paranoid thinking. Biological factors, such as decrease in thalamic volume, have been observed in children with schizophrenia and ultrahigh risk adolescents with associated sleep impairment. Objective studies have indicated possible actigraphy base measures to be the predictor of psychosis after a one year follow-up. The studies using polysomnography have rare and inconsistent results. In this brief review, we provide an overview of existing literature. We also posit that future research will be beneficial in understanding the initiation, course and progression of sleep disturbance in the high risk pediatric population with the goal of implementing interventions to alter the development of psychosis.

## 1. Introduction

Sleep disturbance is considered an important transdiagnostic factor implicated in the development and maintenance of a range of psychiatric disorders [1]. As early as age 4, sleep disturbances, such as difficulty falling asleep, sleep walking, and restless sleep, lead to increased externalizing problems [2]. In adolescents, sleep disorders, such as insomnia, are generally coexistent with internalizing problems like depression and may even predict suicidality [3]. It has been hypothesized that the association between sleep and psychiatric symptoms is often bidirectional and co-occurring sleep or psychiatric disorder impacts the severity of other comorbid disorders [4]. Psychotic disorders are relatively uncommon, yet are some of the most debilitating psychopathologies with onset in late adolescence [5]. The well-formed psychotic disorder, schizophrenia, is characterized by positive symptoms including hallucinations, delusions, disorganization and negative symptoms such as cognitive impairment, apathy, affective flattening, and social withdrawal. There are both objective and subjective studies indicating that a variety of sleep problems ranging from insomnia to parasomnias are associated with psychosis in adults. Certain sleep disorders like insomnia, periodic limb movement disorder, obstructive sleep apnea, and restless leg syndrome are frequently seen in adults with schizophrenia. Presence of significant insomnia has often been observed as a preceding symptom before a psychotic episode and is also shown to impact the prognosis when it continues during the course of illness [5]. There is growing evidence indicating the importance of sleep disorders as potential markers for emergence of psychotic symptoms as well as prognosis of psychotic disorders in adults. However, in the pediatric population, the association between sleep and psychosis is far less researched and not well understood, largely because of the rare prevalence and diagnostic challenges related to ill-defined presentation, convergence of symptom presentation with developmental stages, and difficulty in obtaining reliable chronological history. The purpose of this brief review is to present an overview of the existing literature on the relationship between sleep and psychosis in the pediatric population. 

## 2. Methods

A literature search was conducted in PubMed and Medline databases for original research using search terms “psychosis in children”, “childhood sleep disturbances”, “sleep and psychosis”, “sleep as a predictor of psychosis”, and “insomnia and children” limited to pediatric age range (0–18 years). Both objective and subjective studies published to date in English were included. The titles and abstracts were screened for relevance and the case reports and articles not specifically related to sleep and psychosis in children were excluded. 

## 3. Results

### 3.1. Subjective Studies in Sleep and Psychosis in Children

A multicenter longitudinal study was conducted in North America aimed to identify predictors of psychosis in youth with high psychosis risk [6]. The study results indicated that five features assessed at baseline contributed uniquely to the prediction of psychosis: a genetic risk for schizophrenia with recent deterioration in functioning, higher levels of unusual thought content, higher levels of suspicion/paranoia, greater social impairment, and a history of substance abuse. Sleep disturbance was included in the category of general symptoms, but it did not significantly contribute as a predictor of psychosis. The authors concluded that sleep disturbance accounted for “general symptoms” but did not have any significant predictability factor for psychosis.

A population-based study in United Kingdom that was an offshoot from the second British National survey of Psychiatric morbidity investigated the effect of insomnia [7]. The results determined that sleep difficulties and paranoid fears maintained each other in a circular relationship. Whether the difficulty in falling or staying asleep was mild or severe, the association with paranoia remained; likewise, whether the paranoid thought was of mild or severe content, the association with insomnia was maintained. The results indicated that insomnia was associated with an approximately two to threefold increase in paranoid thinking. The authors concluded that insomnia was a significant contributor to the development and maintenance of paranoid fears. 

A similar UK-based large scale epidemiological study was conducted aiming to investigate the association between specific parasomnias (nightmares, night terrors, and sleepwalking) in childhood and later adolescent psychotic experiences [8] The subjects were recruited from The Avon Longitudinal Study of Parents and Children (ALSPAC) which is a birth cohort study set in the UK. The strength of this study was certainly the sample size which was 4720 individuals. Experience of nightmares, night terrors, and sleepwalking were assessed using a semi-structured interview at age 12. Psychotic experiences were assessed at ages 12 and 18 using a semi-structured clinical interview- Pliksi (Psychosis-Like Symptoms semi-structured interview). Results demonstrated that there was a significant association between the presence of nightmares at age 12 and psychotic experiences at 18 when adjusted for possible confounders and psychotic experiences at 12. As proposed in the study, patients experiencing psychosis may have blurred boundaries between the sleep and awake state and may have brief moments of oscillating between the two, even during the awake state. Nightmares which occur in rapid eye movement (REM) sleep may thus be experienced as hallucinations during the day. Conversely, one could transition into the awake state while asleep, resulting in sleep terrors and sleepwalking in a non-rapid eye movement (NREM) stage. 

A study by Lee et al. investigated the relationship between psychotic-like experiences (PLEs) and sleep disturbances in adolescents [9]. The authors reported that insomnia and excessive daytime sleepiness were found to predict psychotic-like experiences in adolescents independent of depression. The study results indicated that all three types of insomnia (sleep onset, maintenance, and terminal insomnia) predicted high risk of clinical psychosis. Of note, excessive daytime sleepiness was considered to be an independent predictor of PLEs, after controlling for antipsychotic medication, which itself caused sedation.

On similar lines, Taylor et al. tested the hypothesis that shared genetic or environmental influences underlined sleep disturbances and vulnerability to PLEs (psychotic-like experiences) [10]. The study included about 4800 pairs of 16-year-old twins participating in the Twins Early Development Study (TEDS) as part of the Longitudinal Experiences and Perceptions (LEAP) project. The authors concluded that their hypothesis was correct. Three positive symptoms, namely paranoia, hallucinations, and grandiosity, and two negative symptoms of anhedonia and parent -rated negative symptoms were among the PLEs that shared genetic and environmental influences, along with sleep disturbances and cognitive disorganization. This was the only twin study included in our literature search.

Another study examined sleep dysfunction in adolescents at ultrahigh-risk (UHR) for psychosis, relationships between sleep disturbances and psychosis symptoms, volume of an integral sleep-structure (thalamus), and associations between thalamic abnormalities and sleep impairment in UHR youth [11]. In the study, UHR was defined by moderate levels of positive symptoms (unusual thought content/delusional idea, suspicious persecutory ideas, grandiose ideas, perceptual abnormalities/hallucinations, disorganized communication) and/or a decline in global functioning accompanying the presence of schizotypal personality disorder and/or a family history of psychosis [11]. Subjects fulfilling this criterion were included. Conclusively, increased latency to sleep onset and disrupted continuity of sleep were the sleep disturbances observed in UHR adolescents along with decreased bilateral thalamus volume. Interestingly, sleep disturbances in the subjects were associated with greater negative symptom severity as compared to positive symptoms. These findings of specific sleep deficits in UHR youth possibly indicated a likely role for domains of sleep dysfunction (latency, continuity) in the etiology of psychosis. Second, evidence of thalamic reductions in the UHR sample suggested that a critical brain structure supporting sleep function was compromised in adolescents at risk of psychosis. As these abnormalities were present prior to onset, those findings indicated that reductions in sleep-related structures might play a potential role in schizophrenia pathophysiology. 

### 3.2. Objective Studies of Sleep and Psychosis

There has been a very limited number of studies investigating sleep and psychosis using objective measures such as polysomnography and actigraphy in the pediatric age group. A study conducted in 1969 attempted to explore sleep patterns in normal and psychotic children [12]. Considering previous evidence that there was marked decrease in stage 4 sleep in chronic schizophrenic patients, all-night sleep electroencephalograms and power-density configurations were obtained and studied for a group of psychotic and normal prepubertal children. Singular attention was focused on whether stage-4 sleep was affected in the psychotic population who were clearly manifesting thought disorders. Authors reported that there was no significant difference in stage 4 sleep in psychotic versus normal prepubertal children. The authors also concluded that the mean difference of 1 h 37 min in total sleep time between the psychotic and normal children largely reflected the differences in daytime activities between the groups.

More recently, a pilot study was conducted to investigate REM disturbances in children with major depressive disorder (MDD) and schizophrenia [13]. The study concluded that although there was significant REM latency in MDD, it was not seen in children with schizophrenia. However, pronounced impairment of sleep continuity was noted in schizophrenic patients.

In an extension of the above-mentioned subjective study by Lunsford-Avery et al. the authors also obtained the data from actigraphy at baseline and after one year in relation to the psychotic symptoms of ultra-high risk group. The results indicated that the specific sleep measures of actigraphy; reduced duration of sleep, reduced efficiency of sleep, and wake time after sleep onset (WASO), were predictive of the worsening of positive symptoms after a year after controlling for baseline psychosis symptoms [14].

## 4. Discussion

The relationship between sleep disturbance as a predictor of psychosis is not extensively studied in the pediatric population. Sleep research is challenging due to reliability of subjective reports and cognitive abilities affecting self-awareness during different developmental stages in children and adolescents. On the other hand, psychotic disorders are very rare in children and phenomenology can be complex due to the multi-factorial nature of PLEs in that age group, ranging from trauma, mood disorders, maladaptive coping mechanisms to stress, and underlying medical etiologies. 

In the study by Lee et al. [9], the authors concluded that adolescent insomnia was a predictor of PLEs. In contrast, the longitudinal study by Cannon et al. [6], did not support the findings but acknowledged that sleep disturbance is merely a general symptom in youth with risk of psychosis. Although the results of these studies are contradictory, sleep disturbance may have indirectly led to higher levels of unusual thought content or higher levels of suspicion/paranoia in the former study which were considered as predictors of psychosis. It is also important to note that although one study found insomnia to be a predictor of PLE, all PLEs may not eventually progress to psychosis. An interesting finding of the former study is that EDS (excessive day time sleepiness) is an independent predictor for PLE. The authors made an interesting point that narcolepsy having ill-defined boundaries between sleep and wakefulness, as characterized by sleep paralysis and hypnopompic and hypnogogic hallucinations, might be considered as a differential diagnosis of adolescent psychosis and vice versa. 

In the study by Lunsford-Avery et al. [11], the authors stated that problematic sleep might represent a core feature of psychosis, over and above concurrent mood symptoms. However, the offshoot of the same study that used actigraphy measures demonstrated that specific objective parameters at baseline were associated with positive symptoms of psychosis at one year follow-up [14]. On similar lines, the British national survey data indicated specific parasomnias being associated with development of PLEs in future [7]. The authors did attribute the daytime PLEs to the brief lapse into REM sleep. However, while few studies have found poor sleep/wake boundaries among individuals with psychotic symptoms, there is little evidence of intrusions of REM sleep into waking states in patients with schizophrenia experiencing hallucinations.

The twin study by Taylor [10] and study of structural thalamic abnormalities by Lunsford-Avery [11] indicate genetic and biological underpinnings to the association between sleep abnormalities and psychosis. However, the evidence is only limited to association, without any identified direction of effect or causality. 

One of the proposed models to provide a plausible explanation for the bidirectional nature of sleep and psychosis is the neurodevelopmental diathesis-stress model [15]. According to this model, there are shared genetic and environmental factors that affect development of psychosis as well as sleep dysfunction. Additionally, the stress related to psychosocial and biological changes intertwined with neuromaturational factors across developmental stages in children result in the interplay between sleep and cognitive deficits resulting in psychosis like symptoms. While the conceptualization is untested, it draws upon the traditional stress-diathesis model for schizophrenia and also supports the biological underpinnings of sleep and psychiatric disorders. 

Regarding objective studies, the topic has received scant attention and there are very few studies investigating the relationship between objective sleep measures and psychosis. Of the studies described above, only the actigraphy study was longitudinal and reported specific measures to predict psychosis after one year follow-up. However, the rest were mostly limited due to small sample size, different diagnostic criteria, and variability in age groups. Interestingly, the literature in adults indicated that different studies reached different conclusions regarding the objective measures associated with or predicting psychosis. 

## 5. Conclusions

Despite increasing evidence that sleep problems are clinically significant in patients with psychosis and may function as predictors of development of psychosis in individuals with high risk, robust research in this area is lacking. The available literature posits a biological and psychosocial model for explaining the association between sleep dysfunction and psychosis. Large, longitudinal, and objective studies specifically investigating the sleep–psychosis relationship are needed. Further studies should consider replicating some of the findings in adult literature and also investigate the differences between pediatric and adult age groups, thereby providing insights into developmental aspects of psychopathology. It would also be valuable to explore the interventions specific to sleep and cognition as possible ways to alter the development or alleviate dysfunction resulting from psychosis. Finally, it would be important to include sleep symptom assessments in clinical practice as an integral part of assessment and treatment planning for youth presenting with psychosis-like experiences. 
